# Nucleic Acid-Dependent Structural Transition of the Intrinsically Disordered N-Terminal Appended Domain of Human Lysyl-tRNA Synthetase

**DOI:** 10.3390/ijms19103016

**Published:** 2018-10-03

**Authors:** Soon Bin Kwon, Ji Eun Yu, Chan Park, Jiseop Lee, Baik L. Seong

**Affiliations:** Department of Biotechnology, College of Life Science and Biotechnology, Yonsei University, Seoul 03722, Korea; yunbin829@gmail.com (S.B.K.); 4-season-love@hanmail.net (J.E.Y.); justies@naver.com (C.P.); tjqto7@naver.com (J.L.)

**Keywords:** aminoacyl-tRNA synthetase, LysRS, hRID, disorder-helix transition

## Abstract

Eukaryotic lysyl-tRNA synthetases (LysRS) have an N-terminal appended tRNA-interaction domain (RID) that is absent in their prokaryotic counterparts. This domain is intrinsically disordered and lacks stable structures. The disorder-to-order transition is induced by tRNA binding and has implications on folding and subsequent assembly into multi-tRNA synthetase complexes. Here, we expressed and purified RID from human LysRS (hRID) in *Escherichia coli* and performed a detailed mutagenesis of the appended domain. hRID was co-purified with nucleic acids during Ni-affinity purification, and cumulative mutations on critical amino acid residues abolished RNA binding. Furthermore, we identified a structural ensemble between disordered and helical structures in non-RNA-binding mutants and an equilibrium shift for wild-type into the helical conformation upon RNA binding. Since mutations that disrupted RNA binding led to an increase in non-functional soluble aggregates, a stabilized RNA-mediated structural transition of the N-terminal appended domain may have implications on the functional organization of human LysRS and multi-tRNA synthetase complexes in vivo.

## 1. Introduction

Aminoacyl-tRNA synthetases (ARSs) catalyze the aminoacylation of tRNAs, which is an essential step before protein translation can occur [1]. One of these ARSs, lysyl-tRNA synthetase (LysRS), catalyzes bond formation between tRNA^Lys^ and lysine [2]. The three-dimensional structures of *E. coli* LysRS and human LysRS are well-characterized [3,4]. In addition to its canonical function in translation, moonlighting functions of human LysRS including those in the immune response have been recently discovered [5,6]. Eukaryotic LysRSs have an N-terminal appended domain that is absent in prokaryotic LysRSs [7]. This domain is known to non-specifically interact with tRNAs in vitro [7,8], however, the precise role of tRNA binding on structure-functional relationships remains unclear.

Some eukaryotic ARSs also have extensions either at the N or C-terminal extended domain or within the structural domains that are absent in their prokaryotic counterparts [1]. These extensions have high isoelectric points (pI) and the ability to non-specifically bind tRNA [7,9]. The structure of the domains is usually disordered, but adopts a helical structure upon polyphosphate binding [8,10]. This is based on the charge–charge interactions between the negatively charged phosphate backbone and a cluster of positively charged amino acids. However, previous studies have been based on binding to tRNAs and studies about binding with non-specific nucleic acids (NAs) have not been reported. The disordered structure of the extensions without polyphosphate may be induced by the repulsive force exerted by the positively charged cluster, but this has yet to be elucidated.

Recently, the N-terminal RNA-interaction domains (RIDs) of LysRS from eukaryotic origins have been used as novel fusion partners to facilitate folding of their genetically fused cargo proteins [11,12,13,14]. The chaperoning function for all three RIDs—human, rabbit, or mouse (hRID, rRID, or mRID, respectively)—was found to enhance the folding and assembly of down-stream target proteins, while fusion of the mutant hRIDs increased soluble aggregates. The fusion protein was easily purified by a one-step affinity chromatography and treated with a site-specific protease to convert the target protein to its functional form [11,13,14]. Previously, we showed that RNA-binding domains (RBDs) could be used as fusion partners for promoting the solubility and biological activities of target proteins [15]. The results were interpreted as the chaperonic function of RNAs (chaperna; chaperone + RNA) that had been recruited in the vicinity of folding intermediates [16,17,18,19].

As RIDs facilitate the folding and assembly of fused proteins [11,12,13,14], it remains a fascinating possibility that these RIDs may provide chaperoning functions through their interaction with NAs, possibly with tRNAs, for the folding of multidomain ARSs and their subsequent assembly into multimeric complexes [20]. Therefore, the potential role of RNAs in the disorder-order transition would further our understanding of their contribution to the overall folding and assemblage of multi-tRNA synthetase complexes (MSCs) and their canonical and non-canonical functions.

Here, we expressed and purified the RID from human LysRS (hRID) in *E. coli* and also developed mutant hRID constructs which do not bind to NAs. We detected co-purified NAs during the Ni-affinity purification and confirmed that cumulative mutations on critical amino acid residues abolished RNA binding. Due to its unique property of lacking absorbance at 280 nm, we were able to analyze its RNA-binding affinity. We also identified non-specific binding with NAs. Here, we analyzed the secondary structures of the purified proteins, and proved that the repulsive forces in the positively charged cluster destabilized the helical structure and induced a disordered structure. We identified a structural ensemble between the disordered and helical structures for mutants that do not bind RNA, and an equilibrium shift to the helical conformation upon RNA binding. The potential relationships between soluble aggregates and the disordered structure at the N-terminal appended domain may have implications in the functional organization of human LysRS and MSCs in vivo.

## 2. Results

### 2.1. Expression and Purification of hRID and Its Mutants and Detection of Co-Purified NAs

**Figure 1:**
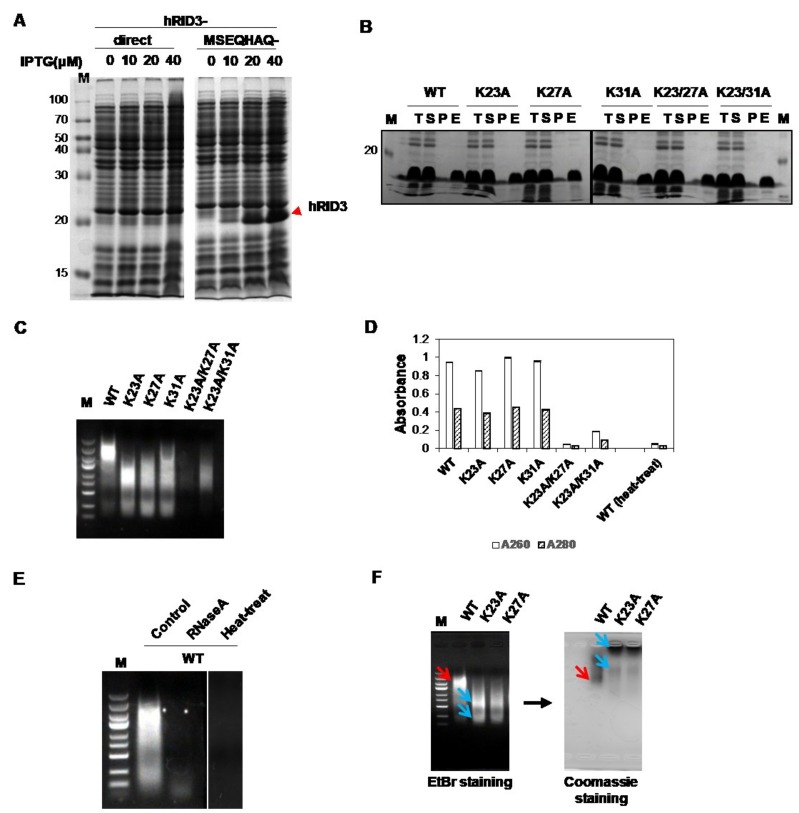
Expression and purification of wild-type and mutant hRID in *E. coli* and confirmation of NA binding ability. (**A**) Recombinant hRID with or without the N-terminal expression-enhancing tag was expressed in *E. coli* by induction with 0–40 μM Isopropyl β-d-1-thiogalactopyranoside (IPTG). Expressed protein is indicated by a red arrowhead. (**B**) Mutant hRIDs were expressed in *E. coli* and purified using Ni-affinity chromatography. T, S, P, and E indicate total lysate, supernatant, pellet, and eluted fraction, respectively. (**C**) Purified proteins were loaded onto an agarose gel and the co-purified nucleic acids (NAs) were detected by EtBr staining. (**D**) Absorbances at 260 and 280 nm of the eluted fractions. The values in the graph represent the means ± standard deviations of the triplicate. (**E**) RNaseA and heat sensitivities of the co-purified NAs were examined. (**F**) Co-purified NAs with hRID (WT, K23A, and K27A) were analyzed by agarose gel electrophoresis, and the gel was stained with EtBr and Coomassie brilliant blue. Colocalized NAs and protein are indicated by the red arrows, and the dissociated NAs and protein are indicated by the blue arrows.

A previous study reported that nine specific positions (K19, K23, R24, K27, K30, K31, K35, K38, and K40) were found to be more important for tRNA binding than the other positions tested [8]. However, the previous study was based on a single-point mutagenesis study of the whole LysRS protein, which have another tRNA-binding domain, so the binding affinity of the mutant hRID alone is unclear. Thus, wild-type hRID (WT) and its mutants (K23A, K27A, K31A, K23A/K27A, and K23/K31A) were expressed in *E. coli* and purified using nickel-charged affinity resin (Ni-NTA) to investigate the binding affinities of hRID and mutant hRIDs. A translational enhancer tag of seven amino acids (MSEQHAQ) was added to the N-termini of the hRID constructs to increase their expression levels (Figure 1A). We found that all proteins could be expressed and well-purified by Ni-chromatography (Figure 1B), and that WT, K23A, K27A, and K31A co-purified with NAs (Figure 1C). However, the amount of co-purified NAs with K23A/K27A and K23A/K31A was decreased, with K23A/K27A having less NAs of the two. The A_260_/A_280_ ratios of the purified NAs were >2.0 (Figure 1D), indicating that most of the bound NAs were RNAs rather than DNAs, because the ratio of pure RNA is 2.0–2.2 and that of pure DNA is 1.6–1.8 [21]. It should be noted that hRID is devoid of the amino acids that are responsible for absorbance at 260 and 280 nm such as Phe, Tyr, Trp, and Cys (Table S1); therefore, it does not interfere with calculating the A_260_/A_280_ scores for determining NA content. As proof of this, the purified double-mutant protein, K23A/K27A, which did not have any co-purified NAs, failed to exhibit UV absorbance at 280 nm (Figure 1D). We also further confirmed that the band was not DNA by examining its heat instability and RNaseA sensitivity (Figure 1E). A greater mobility of tRNA was observed with the single amino acid mutant hRIDs when compared to WT, reflecting reduced affinity (Figure 1C). As proof of this, while WT hRIDs were colocalized with RNAs, dissociation between the mutant proteins and RNAs was evident by agarose gel electrophoresis (Figure 1F). The hRID mutant proteins, K23A and K27A, were mostly localized at the top of the gel, indicating that the mobility was unaffected by the electric field and reflected the neutral net charge of the protein when compared with the negatively charged RNAs. With this data, we concluded that the mutants had a decreased RNA binding ability. The similar levels of co-purified RNA between WT, K23A, K27A, and K31A (Figure 1C,D) were due to excess RNAs in the lysate even though the single point mutants had a lower binding affinity than WT hRID (Figure 1C,F). Interestingly, the apparent molecular weight (MW) of hRID in the gel was two-fold higher than the calculated MW (8.3 kDa). This phenomenon might result from the intrinsic disorder of the hRID structure [22].

**Figure 2:**
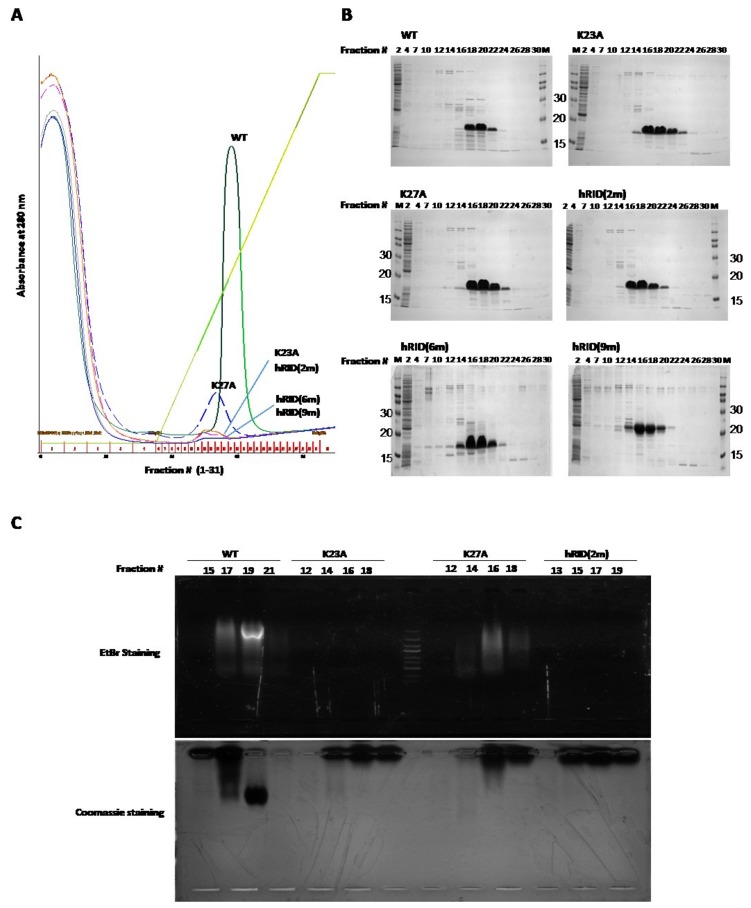
Purification of WT and mutant hRID and detection of them and the co-purified NAs. (**A**) WT, K23A, K27A, hRID(2m), hRID(6m), and hRID(9m) were expressed and purified using an automated purification system and a Ni-affinity column. Co-purified NAs were detected by their absorbance at 280 nm. (**B**) Purified proteins were analyzed by SDS-PAGE. Numbers at the top of the gels indicate fraction number. (**C**) Purified protein and co-purified NAs were confirmed by agarose gel electrophoresis.

Next, we expressed WT, K23A, K27A, and K23A/27A in 400 mL cultures and purified them by automated Ni-chromatography. We also cloned and purified a six-point mutant (K19A/K23A/R24A/K27A/K30A/K31A; hRID(6m)) and a nine-point mutant (K19A/K23A/R24A/K27A/K30A/K31A/K35A/K38A/K40A; hRID(9m)). Although absorbance is usually measured at 280 nm to detect purified protein, hRID does absorb 280 nm light (Table S1). Thus, the purified proteins were analyzed on sodium dodecyl sulfate polyacrylamide gel electrophoresis (SDS-PAGE) and their absorbances at 280 nm were used to detect the co-purified NAs. We found that all proteins were well-purified. Furthermore, hRID and K27A co-purified with RNAs, while hRID(6m) and hRID(9m) did not (Figure 2A,B). We also detected a small amount of co-purified NAs with K23A and K23A/K27A (henceforth called hRID(2m)) (Figure 2A). Co-purified RNAs were confirmed by agarose gel electrophoresis with EtBr staining (Figure 2C). Even though K23A and K23A/K27A co-purified with NAs, their concentration was too low to be detected by EtBr staining (Figure 2C). Unlike the results obtained by manual purification (Figure 1B), the amount of RNA that co-purified with K23A and K27A by automated chromatography was lower than that of WT hRID due to the flow-wash of the automated system.

### 2.2. Separation of the Proteins with Co-Purified NAs

**Figure 3:**
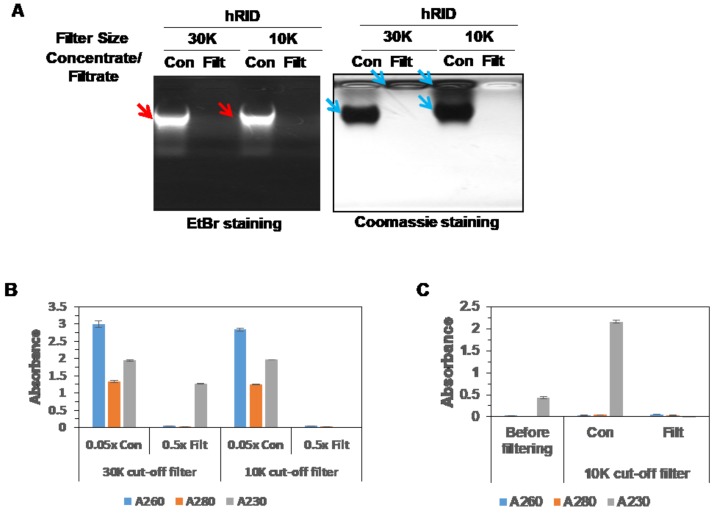
Purification of NA-free protein. (**A**) Purified hRID protein was tested for the removal of RNA after centrifugation through either 30K or 10K cut-off filters. Concentrated samples (Con) and filtrated samples (Filt) were analyzed by agarose gel electrophoresis. RNAs and proteins are indicated with red and blue arrows, respectively. (**B**) Absorbances of the samples in (**A**) at 260, 280, and 230 nm with the proper dilution. (**C**) NA-free hRID was concentrated using a 10K cut-off filter, and the absorbances at 260, 280, and 230 nm of 6-fold diluted samples were measured. The values in each graph represent the means ± standard deviations of the triplicate.

The RNA that co-purified with WT hRID was found even after the dialysis and concentration steps. Since RNA-free hRID was needed, and RNase could not be used, we separated the hRID protein and co-purified RNA using a concentrator kit with 10K and 30K cut-off filters (Figure 3A). Macromolecules bigger than the filter pore size did not penetrate the filter, while those smaller than the pores did. hRID protein is quite small—approximately 9 kDa—and we hypothesized that the co-purified RNAs were larger than the hRID protein (a 27 nt RNA = ~9 kDa). When using the 10K cut-off filter, both the protein and RNA were unable to pass. Even though free hRID is smaller than 10K, it could not penetrate the filter. This may be because hRID has a disordered structure, so it acts like a larger protein. However, using the 30K cut-off filter, we were able to obtain pure protein without RNA, since only the protein passed through the filter. The absence of NAs was confirmed by the absence of absorbance at 260 and 280 nm, and the presence of protein was confirmed by the absorbance at 230 nm, an indicator of peptide bonds (Figure 3B). The high absorbances at 230 nm of the concentrated samples was due to the presence of NAs as they also absorb at 230 nm. After RNA elimination, the protein was concentrated using a 10K Centriprep (Figure 3C). We further confirmed the oligomeric state of the purified hRID by size-exclusion chromatography (Figure S1). The apparent MW of unbound hRID was 27.64 kDa, which may suggest that hRID is a trimer. However, a previous study of purified hRID found that it was monomeric, and that the higher MW was due to its disordered structure [7].

### 2.3. Binding Affinity of hRID and Its Mutants

**Figure 4:**
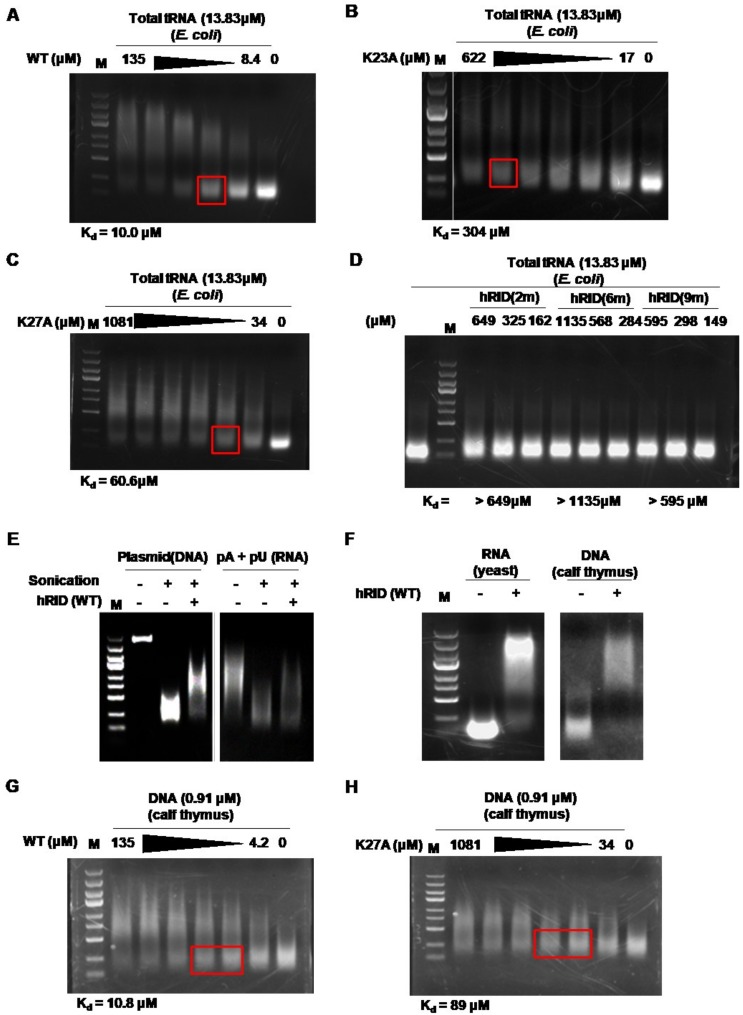
hRID nonspecifically interacts with NAs. (**A**–**D**) The interactions between total tRNAs from *E. coli* and (**A**) hRID, (**B**) K23A, (**C**) K27A, (**D**) hRID(2m), hRID(6m), or hRID(9m) were confirmed using an electrophoretic mobility shift assay (EMSA). The point where half of the tRNAs were bound to the proteins was marked by a red box, and the dissociation constant (K_d_) was calculated. (**E**) Plasmid DNA and poly(A/U) were sonicated and analyzed by EMSA using WT hRID. (**F**) Total RNAs from yeast and total DNAs from calf thymus were fragmented by sonication, and binding to hRID was tested. (**G**,**H**) The interaction between sonicated DNA from calf thymus and (**G**) hRID or (**H**) K27A was confirmed by EMSA. A red box indicates where half of the DNAs were bound to the protein.

The tRNA-binding affinity of hRID was analyzed by an electrophoretic mobility shift assay (EMSA). As shown in Figure 4A, the mobility of the tRNA was dependent on the concentration of WT hRID. The dissociation constant (K_d_) was calculated based on the ratio of bound to unbound forms, and measured values represent the average K_d_ of each tRNA in total tRNAs. The K_d_ for binding of total tRNAs to WT hRID was estimated to be 10 µM (Figure 4A). In contrast, the K_d_ values for the K23A or K27A mutants were 304 and 60 µM, respectively, reflecting reduced affinities (Figure 4B,C). The K_d_ values for hRID(2m), hRID(6m), and hRID(9m) were at least 60-fold higher than WT and were too high to be measured under our experimental conditions (Figure 4D).

### 2.4. hRID Can Bind to NAs Non-Specifically

Non-specific binding between hRID and tRNA is based on the clusters of positively charged amino acids in the protein and the negatively charged phosphate backbone of the tRNA [8]. As a result, we predicted that hRID could also bind to the backbone of RNA or DNA. Previous reports have described that the N-terminal appended domains of other tRNA synthetases such as yeast AspRS can interact non-specifically with NAs [10,23]. However, there have only been reports on hRID to tRNAs and not to other NAs [7,8,11,13]. Here, we examined the binding between hRID and DNA (pGE hRID plasmid) or poly(A/U) RNA. We initially failed to observe a mobility shift with NAs, probably due to the relatively small effects that large NAs have on retardation (data not shown). Thus, DNA and RNA were fragmented by sonication before EMSA. As previously reported [24], the fragments retained a lower size-limit after prolonged sonication. After sonication, however, the mobility shift was markedly enhanced (Figure 4E). The fragmented, purified total DNAs and RNAs from calf thymus and yeast, respectively, were also retarded by hRID (Figure 4F). The K_d_ values of WT and K27A with sonicated calf thymus DNA were quite similar to those with tRNA (Figure 4G,H). Taken together, these results clearly show that hRID can interact with a variety of NAs including tRNAs with tertiary structure, homopolymeric poly(A/U), and DNA. However, the co-purified NAs with hRID were biased towards being RNAs (Figure 1E), probably due to there being a higher (~10-fold) concentration of RNAs than DNAs in the growing *E. coli* cells [25] as well as the higher structural constraints imposed on genomic DNA.

### 2.5. Secondary Structure of WT and Mutant hRID Constructs

Previous reports have shown that the charge-rich regions (from 19 to 40 amino acids) of RIDs are disordered but adopt helical structures upon binding to NAs [8,10]. When RIDs obtain their helical structure, positively charged amino acids are clustered to bind to the negative charge of the NAs [8]. Likewise, NAs can induce this conformational change of RID. In other words, the aligned positively charged amino acids are important for NA binding and helical structure formation. Conversely, we speculated that the aligned, positively charged amino acids would create a repulsive force, thereby destabilizing the helical structure in the absence of NAs. Thus, we performed secondary structure prediction and circular dichroism to compare the WT and mutant hRIDs.

**Figure 5:**
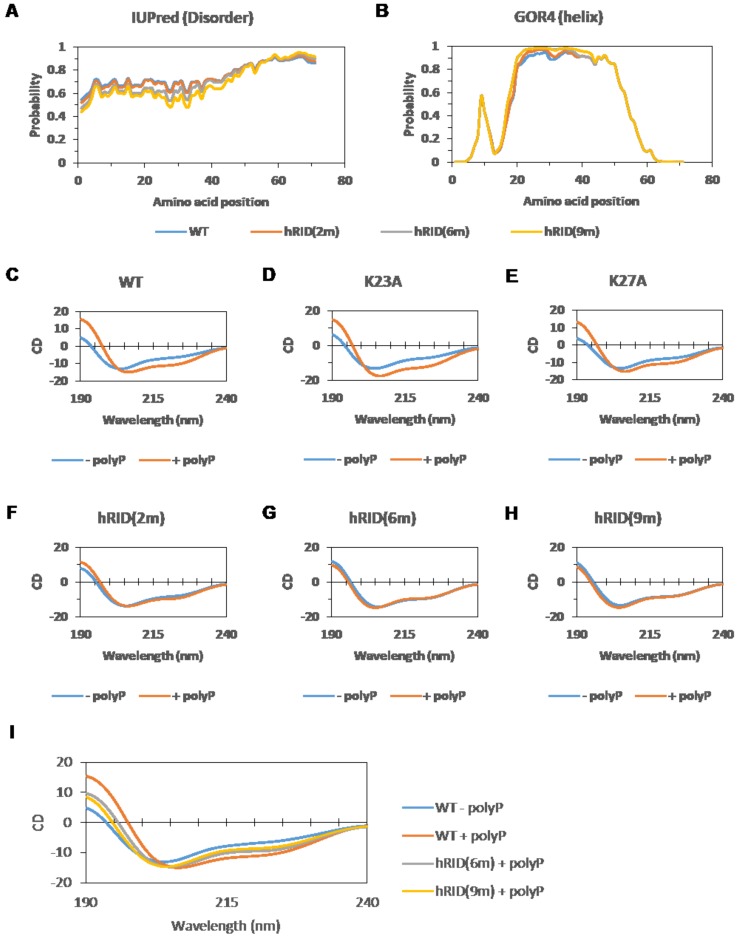
Prediction and investigation of the secondary structure of WT and mutant hRIDs. (**A**,**B**) Prediction of (**A**) disordered regions and (**B**) helical regions of WT or mutant hRIDs. (**C**–**H**) Circular dichroism spectra of (**C**) WT, (**D**) K23A, (**E**) K27A, (**F**) hRID(2m), (**G**) hRID(6m), or (**H**) hRID(9m) with (−) or without (+) polyphosphate (polyP). (**I**) Comparison of the circular dichroism spectrum of “‘hRID(6m) + polyP’ or ‘hRID(9m) + polyP’” with Figure (**C**).

The tendency of adopting disordered or helical structures in WT and mutant constructs was predicted using disorder and secondary structure predictors (Figure 5A,B). There was only a small difference between them, and the region from 19 to 40 was predicted to have a high probability for being both disordered and helical.

Next, we examined the secondary structure of WT and mutant hRIDs by circular dichroism (Figure 5C–H). We used polyphosphate (polyP) rather than RNA or DNA as purines and pyrimidines (A, T, G, C, and U) result in high noise during circular dichroism. We found that the structures of WT, K23A, and K27A changed upon polyP addition (Figure 5C–E). Upon polyP addition, the circular dichroism (CD) score at 225 nm was decreased, and the CD score at 190 nm was increased, indicating that helical content increased. However, no significant changes were observed in the structures of hRID(2m), hRID(6m), or hRID(9m) after polyP addition (Figure 5F–H). These results correlate with the RNA binding affinities of WT and mutant hRIDs (Figure 1, Figure 2 and Figure 4).

**Figure 6:**
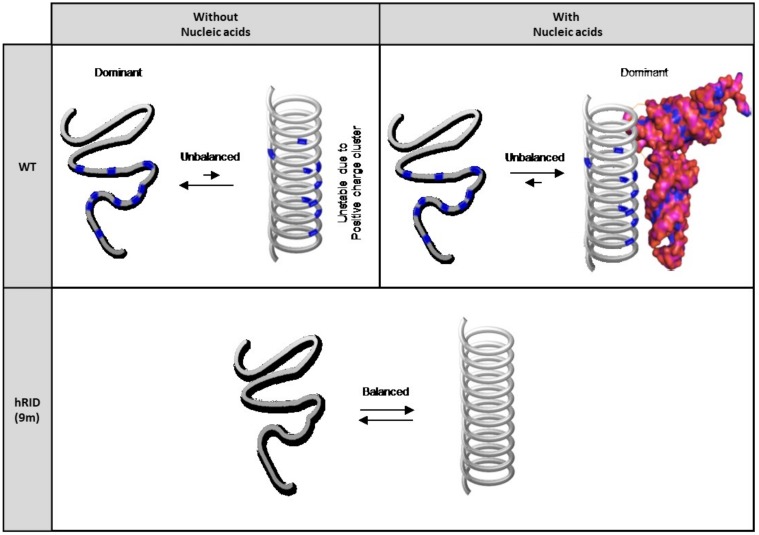
Diagram representing the structural equilibrium of WT and hRID(9m). WT hRID has an unbalanced equilibrium state. Intrinsically, WT has a disordered structure, which turns helical upon NA binding. However, hRID(9m) has a balanced equilibrium state regardless of the presence of NAs.

We then compared the circular dichroism results of hRID(6m) and hRID(9m) with that of WT (Figure 5I). The results of hRID(6m) and hRID(9m) were in-between the results of WT with and without polyP, suggesting that WT hRID without NAs has a less helical content than hRID(6m) or hRID(9m). This also suggests that the helical structure of WT hRID alone is less stable than that of mutant hRIDs, which may be due to the repulsion of positively charged amino acids. However, with NAs, the clusters of positive charges can be stabilized by the negatively charged backbone of polyP, wherein the helical structure is dominant (Figure 6).

## 3. Discussion

In addition to their conserved role in translation, some ARSs including LysRS have additional domains with unique structural characteristics that are neither part of the enzymatic core nor present in bacterial homologs [1,7]. The newly evolved domains are not essential for tRNA charging, but rather are responsible for the non-canonical, moonlighting activities unrelated to aminoacylation [26]. Nevertheless, defects in either canonical or non-canonical ARS functions are associated with human diseases [27].

The N-terminal appended domain of human LysRS (hRID) is intrinsically disordered and known to interact with tRNAs in vitro, but the precise role of tRNA binding on the structure-functional relationship remain unclear. Here, we performed a detailed mutagenesis analysis on hRID, focusing on the pivotal residues associated with tRNA binding. The affinity to tRNAs was greatly reduced by the single point mutations, K23A and K27A, with a 30-fold and 6-fold increase in their dissociation constants, respectively. The combinations of mutations—double, six, or nine-point mutants—further increased the dissociation constant by more than 60-fold (Figure 4). While K23A/K27A still had a weak, but detectable NA binding ability (Figure 2A), all of the multiple mutants—hRID(6m) and hRID(9m)—failed to exhibit affinities to RNAs (Figure 2A and Figure 4D). We developed a simple method to separate the hRID protein from co-purified RNAs (Figure 3). Here, we confirmed that hRID could non-specifically bind to any NA (Figure 4) and was not restricted to tRNAs. The non-specific nature of the binding is most likely mediated by the positively charged amino acids of hRID and the negatively charged phosphate backbone of the NAs. The secondary structure examined by CD in the presence of polyphosphates suggests that the positively charged amino acids in hRID are involved in the conformational change from disorder to an alpha helix in the presence of NAs (Figure 5 and Figure 6). Importantly, further extending previous observations [10], we identified a structural ensemble between the disordered and helical structures in the mutants. For the WT construct, however, the equilibrium shifted in favor of the helical conformation in the presence of polyphosphates.

The major structural domain of LysRS is composed of anticodon binding and aminoacylation domains, and the proper folding of these domains is essential for the aminoacylation of tRNA^Lys^—its canonical function in protein synthesis. Previously, it was shown that the N-terminal domains of native multidomain proteins have the potential to assist with de novo folding of their downstream domains in vivo by acting as solubility enhancers [28]. As such, the N-terminal domains of multidomain proteins of *E. coli* origin such as LysRS are potent solubility enhancers for various C-terminal heterologous proteins [28]. Importantly, tRNA increased the refolding yield of prokaryotic LysRS in vitro [15], and is direct proof of tRNA-dependent chaperone activity. Likewise, the hRID, at the extreme N-terminal appendage of human LysRS, which is absent in the *E. coli* counterpart, may serve as a *cis*-acting chaperone for the folding of downstream structural domains of human LysRS. Notably, when hRID is fused to the N-termini of target antigen proteins, RNA binding to hRID potently stimulates the folding and assembly of antigens into immunologically relevant multimers, virus-like particles (VLPs), or nanoparticles [13,14].

The use of mutant hRID constructs verified that NAs function as *trans*-acting factors, which, by binding to hRID, harness hRID as a solubility enhancer for the downstream domains [13,14]. When fused to aggregation-prone proteins, the non-RNA-binding mutant fusions were present predominantly as inactive soluble aggregates of ill-defined structures, whereas WT fusion forms were present as regular assemblages [13,14]. Consistent with the role of disordered domains as high solubility enhancers [29,30], the non-RNA-binding mutants with disordered structures had increased solubility. However, this represents an increase in soluble aggregates rather than proper folding, confirming that RNA-binding is crucial for its folding into a biologically relevant conformation. These results suggest that NA binding is important for the structural stabilization of the N-terminal appendage, and the disorder-to-order transition at the N-terminal domain may be required for the structural maintenance of LysRS so that it can serve its canonical function in aminoacylation as well as its non-canonical, moonlighting activities [6,31,32,33]. These results are consistent with the chaperoning role of the N-terminal domains of native multidomain proteins in assisting de novo folding of their downstream domains in vivo [28,34]. It should be emphasized, however, that here, the chaperoning role of N-terminal domains is exerted in concert with the bound RNAs [13,14,15,16,17,18,24,35,36].

We assumed that hRID would maintain a helical structure in a cellular environment due to the high concentration of NAs. Considering that the proper folding of LysRS is a prerequisite for its assembly into MSCs [20], it is tempting to suggest that tRNA binding to de novo synthesized N-terminal domains controls the folding and assembly pathways of MSCs. This interpretation is consistent with the observation that tRNA binding to hRID induces the folding and assembly of fused proteins [13,14]. A recent paper also showed that the folding and assembly of cellular macromolecules are controlled cotranslationally by binding to some subunits of the complex during translation [37], supporting the idea that RNA binding to hRID facilitates the folding of hLysRS and assembly of MSCs.

Moreover, tRNA binding may be potentially related to its quality control for performing non-canonical functions in various cellular compartments or after secretion out of the cell [5,38]. hLysRS is present not only in cytosol, but also in the nucleus, mitochondria, plasma membrane [39], and hRID is critical for nuclear localization of hLysRS [39]. In our bacterial expression system, the majority of NAs that co-purified with hRID were RNAs (Figure 1C,E); although, hRID was also able to bind to DNA (Figure 4). Preferential binding to RNA might be due to the higher molar concentration of RNAs in growing *E. coli* cells [25], but whether DNA binding represents in vitro artifacts or has biological significance remains to be further studied. In the eukaryotic cytosol, hRID has little chance to interact with DNA, which is enclosed in the nucleus. It should be noted, however, that hLysRS can relocate to the nucleus [39], and due to the high DNA concentration in nucleus, the hRID of hLysRS may be involved in DNA binding. Whether this activity is associated with the activation of transcriptional factors by hLysRS subsequent to its nuclear translocation requires further investigation [40]. hLysRS also functions, as part of the innate immune system as a cytokine to trigger proinflammatory responses [38,41]. Accumulating evidence suggests that the structures and functions of disordered proteins are largely influenced by the physicochemical properties of their cellular environments [42]. Whether RNA-dependent structural modulation of hLysRS is involved in its non-canonical functions in various intracellular organelles, the secretion process, or after secretion into the NA-devoid extracellular environment merits further investigation.

In conclusion, the stabilized, RNA-mediated structural transition of the N-terminal appended domain in vitro has implications on the functional organization of human LysRS into MSC in vivo, both for its canonical function in translation and for its non-canonical moonlighting activities. This study usefully guides the design of chaperna (RNA-mediated chaperone) for the folding and assembly of target proteins of interest for biomedical applications.

## 4. Materials and Methods

### 4.1. Expression Vector Construction

The pGE-hRID3 vector was constructed from the pGE-LysRS vector [15]. The pGE-LysRS and the PCR products of hRID were cleaved with NdeI/HindIII, and the PCR product was inserted into the cleaved pGE vector. Expression vectors for mutant hRID were constructed using the Dokdo™ Site-Specific Mutagenesis Kit in accordance with the manufacturer’s instructions (Elpis-biotech, Daejeon, South Korea; Cat. No. EBT-5001). To ensure high protein expression, 5′-proximal sequences of eLysRS (ATGTCTGAACAACACGCACAG-, corresponding to the amino acid sequence, MSEQHAQ) were added at the 5′-ends of all hRID genes as a translational enhancer [15,43].

### 4.2. Protein Expression and Purification

BL21*(pLysS) were transformed with each expression vector, and the transformed cells were grown in 3 mL of LB medium with ampicillin (50 μg/mL), or with ampicillin (50 μg/mL) and chloramphenicol (34 μg/mL) overnight at 37 °C. A total of 0.2 mL of each culture was inoculated into 4 mL of fresh LB medium with the same antibiotics, followed by growth at 37 °C until OD_600_ > 0.5. Then, 1 mM IPTG was added, and the cells were grown at 37 °C. After 3 h of incubation, the cells were harvested, suspended in PBS, sonicated, and fractionated into soluble proteins (S) and insoluble proteins (P, pellet) by centrifugation. For experiments requiring pure protein, target proteins in the soluble fraction were purified using HisPur™ Ni-NTA Resin (Thermo Fisher Scientific, Waltham, MA, USA; Cat. No. 88221) in accordance with the manufacturer’s instructions. Each fraction (total lysate (T), S, P, and the elution (E)) was analyzed by SDS-PAGE. The amount of target protein was estimated by densitometry analysis of the stained gel using Bio1D (VILBER). Nucleic acids (NAs) that copurified with hRID in the eluted samples were analyzed by agarose gel electrophoresis in pH 8.3 TAE buffer (Biosesang, Seongnam, Korea; Cat. No. T2002) and the absorbances at 260 and 280 nm. To verify the identity of the co-purified NAs, purified hRID was either treated with RNaseA or incubated at 45 °C for 30 min.

### 4.3. Large Culture and Purification

First, BL21*(pLysS) competent cells were transformed with the pGE-hRID vector. Transformed cells were grown in 3 mL of LB media with ampicillin (50 μg/mL) and chloramphenicol (34 μg/mL) for about 8 h at 37 °C. Then, 0.1 mL of the media culture was inoculated into 50 mL of fresh LB media with the same antibiotics, and grown overnight at 37 °C. After incubation, 20 mL of cultured cells was poured into 400 mL of fresh LB media with the same antibiotics. This was grown at 37 °C until OD_600_ > 0.7, at which point 1 mM IPTG was added to induce hRID protein expression and the culture was incubated for 4 h at 37 °C. The cells were harvested and stored at −80 °C.

To purify hRID, the harvested cells were lysed by sonication in 10 mL of buffer A (50 mM Tris-Cl, pH 7.5, 300 mM NaCl, 10% glycerol, 10 mM imidazole, 0.05% Tween 20, and 2 mM β-mercaptoethanol). Soluble lysate was obtained by centrifugation at 15,000× *g* for 12 min and filtered using a 0.45 μm syringe filter (HYUNDAI Micro, Seoul, Korea; SP25P045S). hRID was purified using an Äkta prime plus (GE Healthcare Life Science, Chicago, IL, USA) equipped with a HisTrap HP column (GE Healthcare Life Sciences, 17-5248-02) that had been equilibrated with buffer A. hRID was eluted with a continuous gradient of buffer B (same composition as buffer A, except with 300 mM imidazole). The purified protein was detected by SDS-PAGE and co-purified NAs were identified by agarose gel electrophoresis and absorbance at 280 nm. Fractions containing purified protein were collected and dialyzed against storage buffer (PBS, 0.05% Tween 20, and 2 mM β-mercaptoethanol) using Spectra/Por-1 or -4 Dialysis Trial Kits (Spectrum Labs, Waltham, MA, USA; Cat. No 132650 and 132700, respectively). Then, the samples were concentrated using 10K or 30K Centriprep filters (Millipore, Burlington, MA, USA; Cat. No 4304 and 4306, respectively). Both the concentrated and filtrated samples were analyzed by agarose gel electrophoresis, and the absorbances at 260 and 280 nm were measured to detect NAs. The absorbance at 230 nm was measured to detect protein. The RNA-free hRID, which was the filtrate of the 30K Centriprep membrane, was re-concentrated using a 10K Centriprep membrane. SDS-PAGE gel was stained with Coomassie brilliant blue R-250. The agarose gel was stained with EtBr and Coomassie brilliant blue R-250.

### 4.4. Electrophoresis Mobility Shift Assay

RNA-free hRID was mixed with various types of NAs including total tRNA from *E. coli* (Sigma-Aldrich, St. Louis, MO, USA; Cat. No. R-1753), pGE backbone plasmid DNA, polyA + polyU mixture (Pharmacia Biotech, Township, NJ, USA; Cat. No. 27-4110-01 and 27-4440-02, respectively), calf thymus DNA (Sigma-Aldrich; 27-4563-01), and yeast RNA (Sigma-Aldrich; R-6750). Each sample, except for the total tRNA, was fragmented by sonication for better resolution in the electrophoresis mobility shift assay (EMSA). Total tRNA and fragmented NAs were mixed with the defined amount of hRID, incubated for 10 min at room temperature, and analyzed by agarose gel electrophoresis.

### 4.5. Prediction of the Intrinsically Disordered Regions and the Protein Secondary Structure

The disorder tendency and the secondary structure of hRID and mutant hRIDs were predicted using IUPred [44,45] and GOR IV [46], respectively.

### 4.6. Circular Dichroism

The hRID that had been purified by Ni-affinity chromatography was dialyzed against 10 mM phosphate (pH 7.5). Then, 8.8 μM hRID was mixed with or without 50 mM P100 polyphosphate (polyP) (Kerafast, Boston, MA, USA; Cat. No. EUI005), and the CD spectra of the hRID-polyP mixtures were recorded using 1-mm cells. For CD measurements, a Jasco 700 CD spectrophotometer was used under 10 psi nitrogen conditions with a step resolution of 1 nm and a scan speed of 50 nm/min. Here, 10 mM phosphate was used as a baseline, and all spectra were scanned three times and smoothed using Spectra Manager (Jasco, Easton, MD, USA).

## Supplementary Materials

Supplementary materials can be found at http://www.mdpi.com/1422-0067/19/10/3016/s1.
