# Supplementary material for: Nucleic Acid-Dependent Structural Transition of the Intrinsically Disordered N-Terminal Appended Domain of Human Lysyl-tRNA Synthetase
Source: Int J Mol Sci. 2018 Oct 3;19(10):3016. doi: 10.3390/ijms19103016 (PMC6213541; doi:10.3390/ijms19103016)
Supplement: Supplementary file 1 [file ijms-19-03016-s001.pdf]

Table S1. Amino acid composition of hRID. Amino acids required for absorbance at 260 nm and 280 nm are marked in red.

Type	Amino Acid	# of Residues
Non-polar	Ala	11
	Val	6
	Leu	5
	Ile	0
	Pro	2
	Met	0
	Phe	0
	Trp	0
Polar	Gly	3
	Ser	5
	Thr	4
	Cys	0
	Asn	3
	Gln	4
	Tyr	0
Negatively charged	Asp	2
	Glu	11
Positively charged	Lys	11
	Arg	2
	His	1

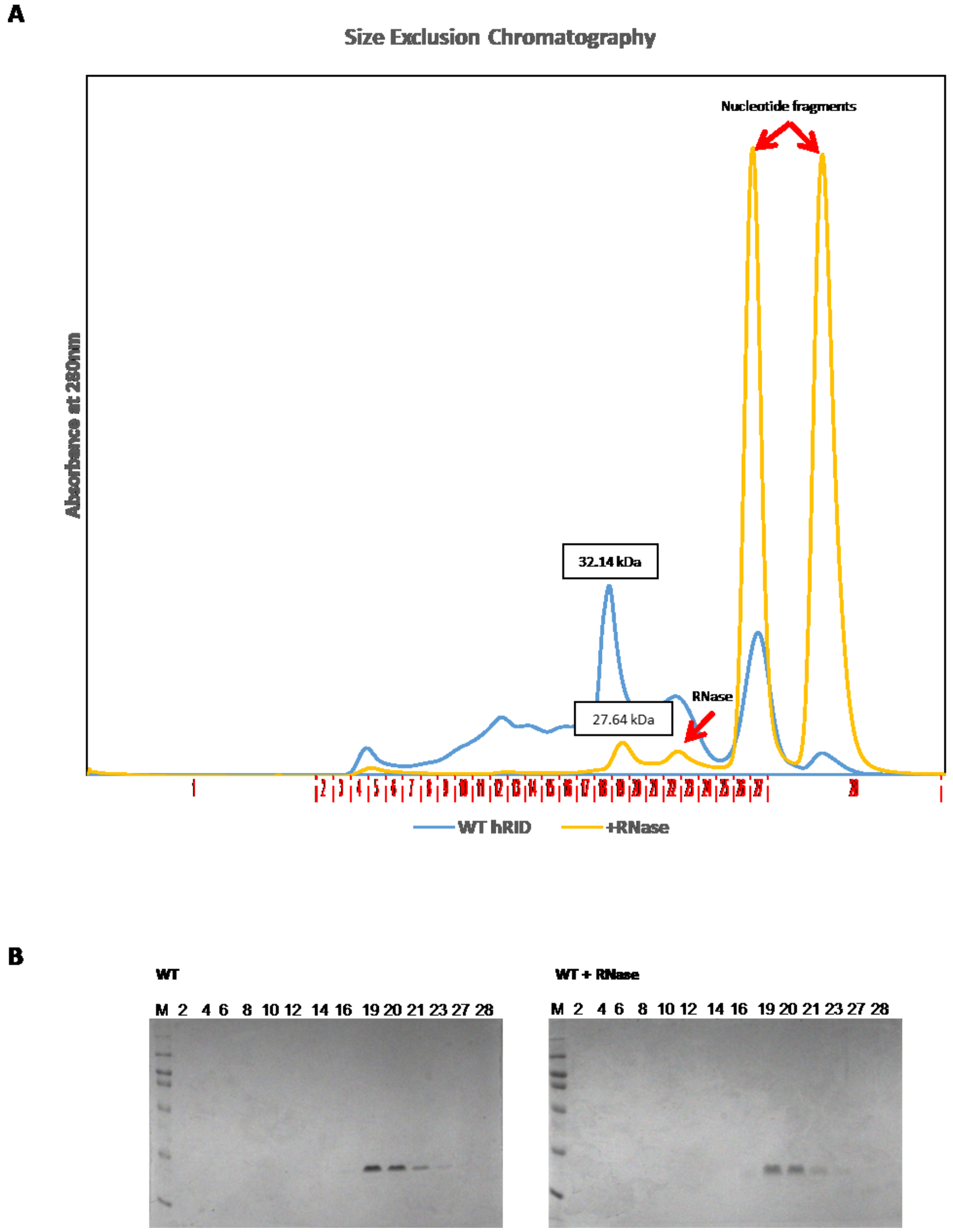

Figure S1. Purified hRID is disordered and in the monomeric state. (**A**) Size-exclusion chromatography was performed to verify the quality of purified hRID and RNaseA treated hRID. (**B**) Absorbance at 280 nm was very low; therefore, the presence of the protein was confirmed by SDS-PAGE. The calculated molecular weight (MW) of hRID have been shown in black boxes.
